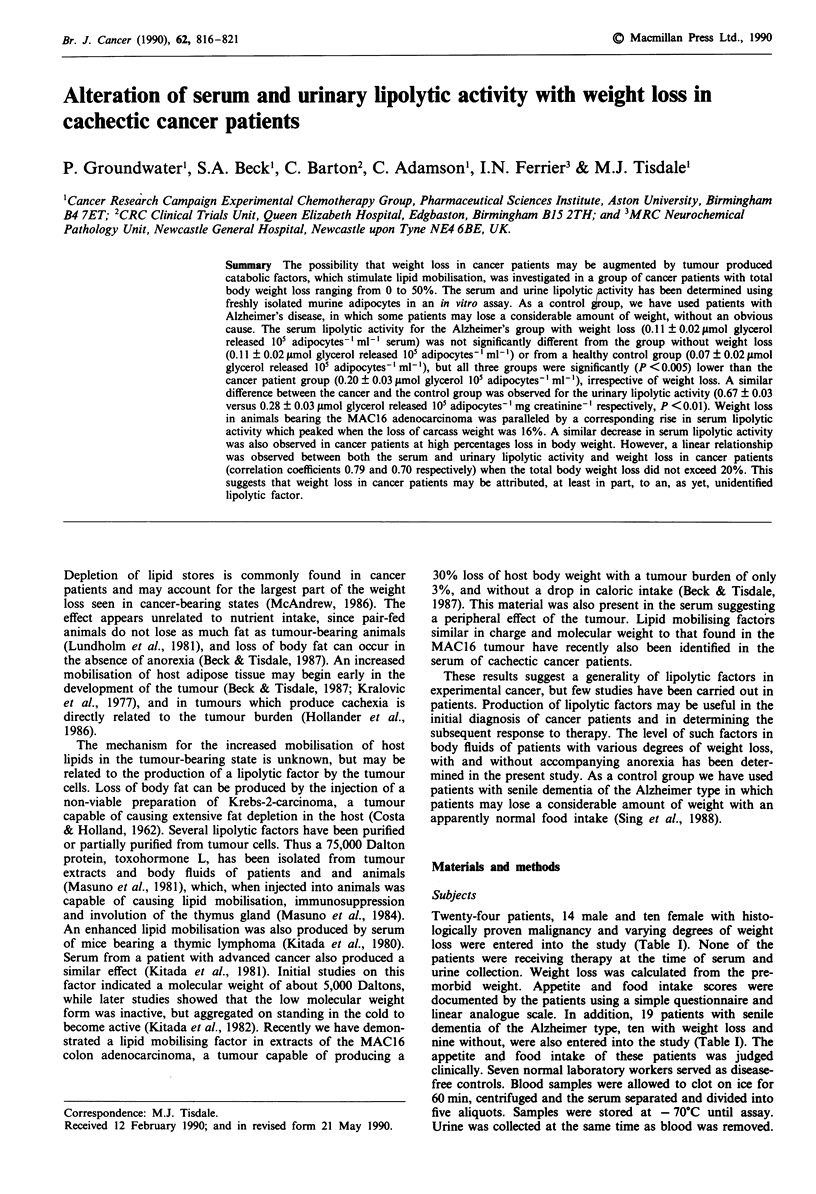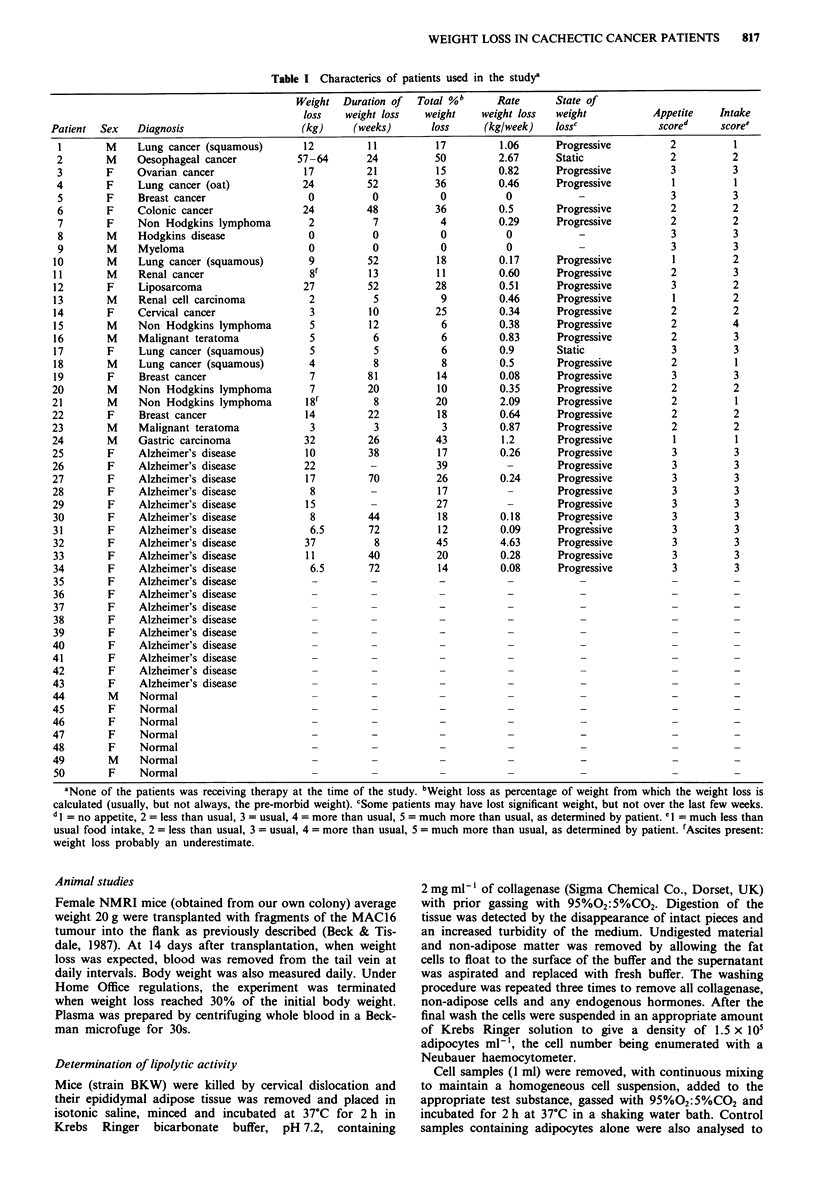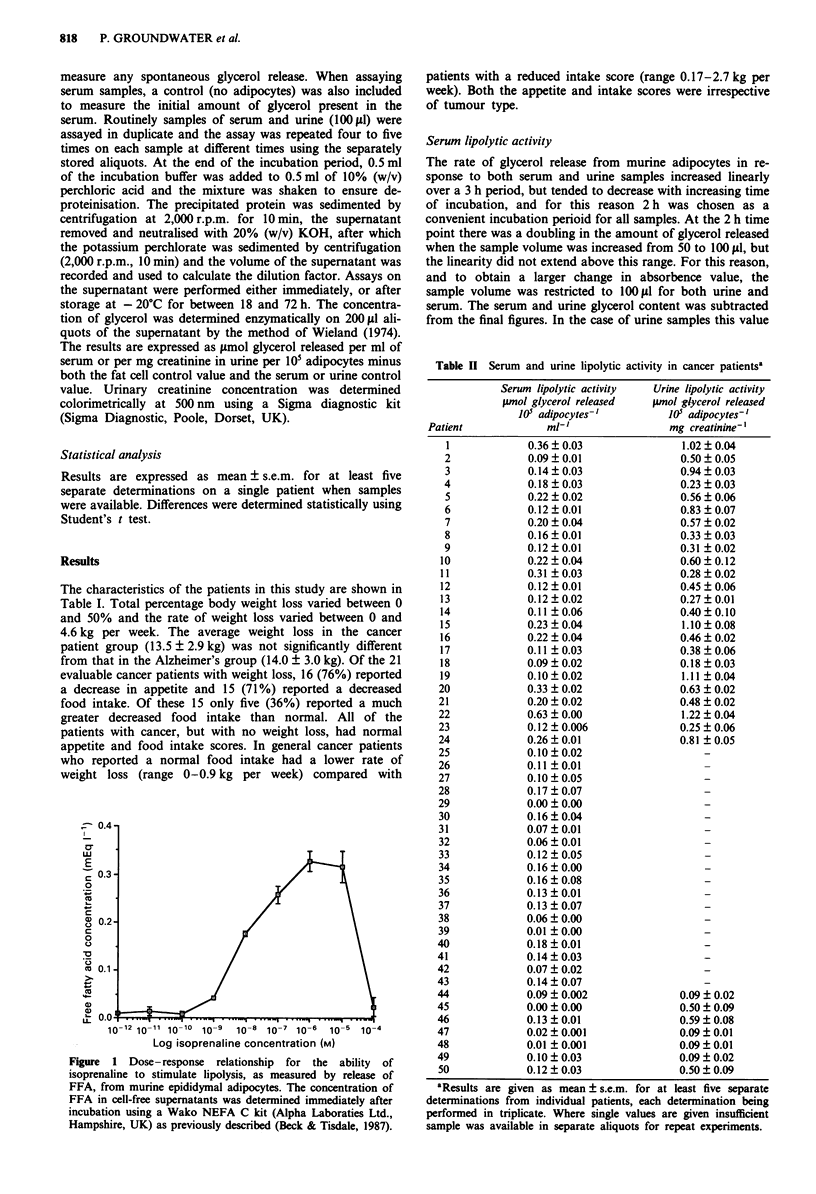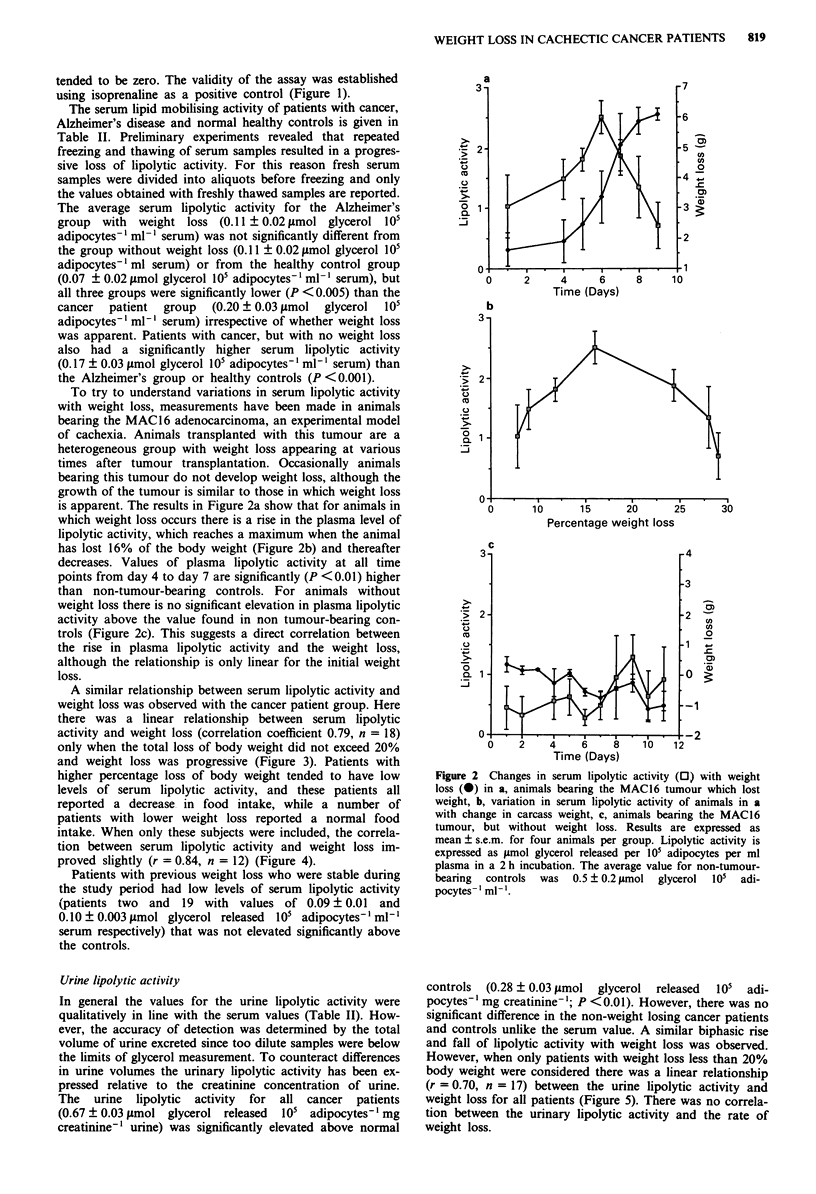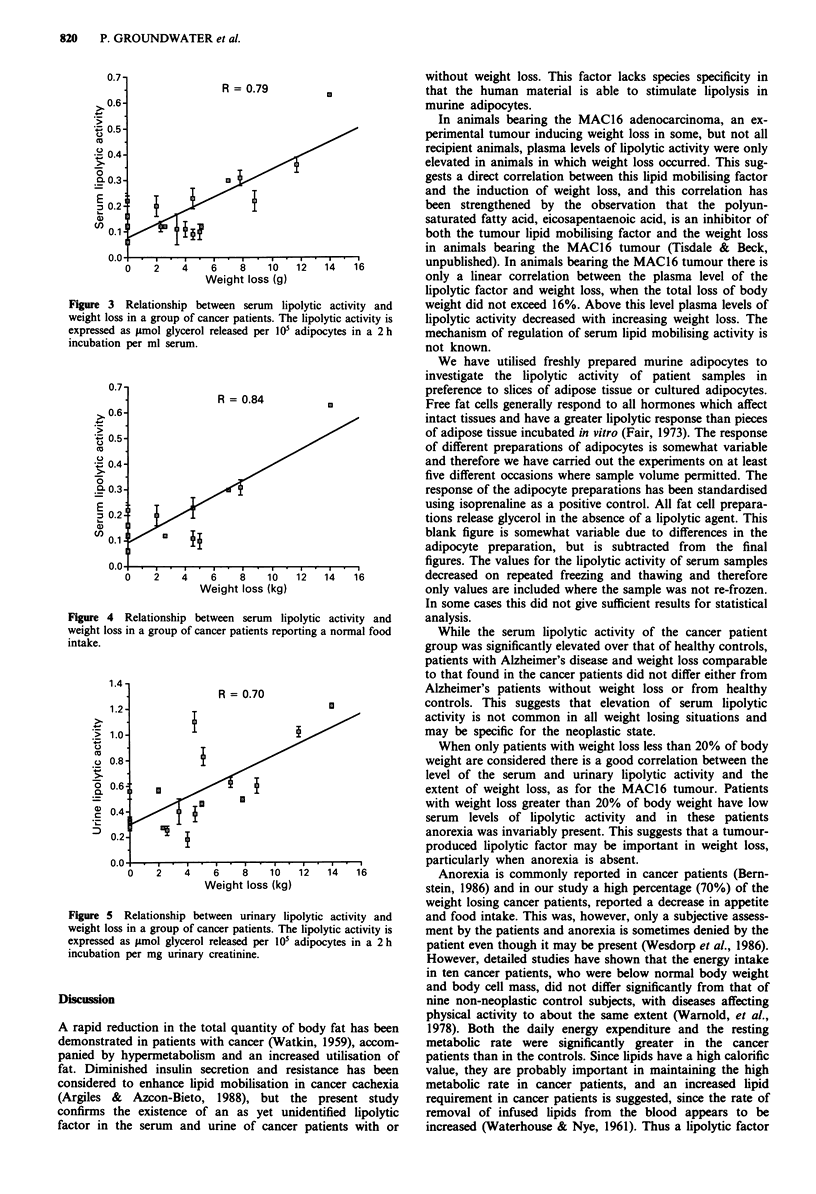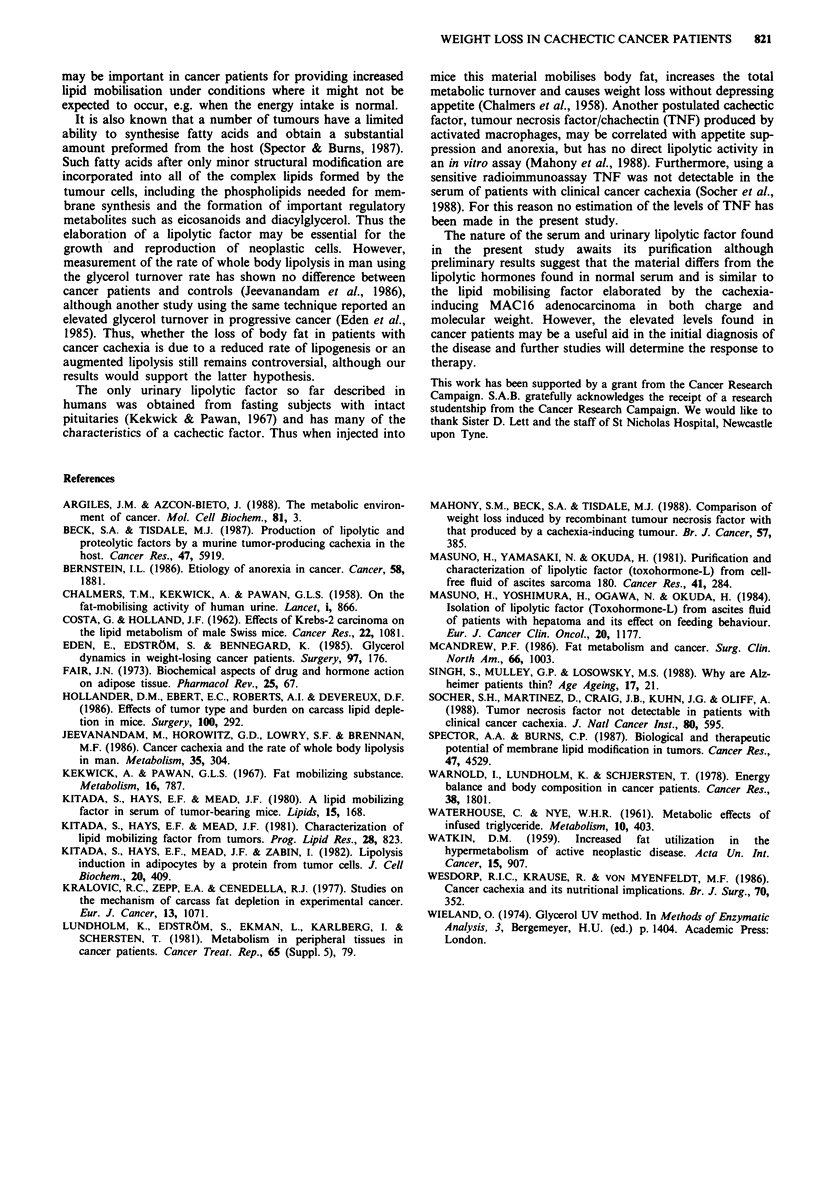# Alteration of serum and urinary lipolytic activity with weight loss in cachectic cancer patients.

**DOI:** 10.1038/bjc.1990.384

**Published:** 1990-11

**Authors:** P. Groundwater, S. A. Beck, C. Barton, C. Adamson, I. N. Ferrier, M. J. Tisdale

**Affiliations:** Cancer Research Campaign Experimental Chemotherapy Group, Pharmaceutical Sciences Institute, Aston University, Birmingham, UK.

## Abstract

The possibility that weight loss in cancer patients may be augmented by tumour produced catabolic factors, which stimulate lipid mobilisation, was investigated in a group of cancer patients with total body weight loss ranging from 0 to 50%. The serum and urine lipolytic activity has been determined using freshly isolated murine adipocytes in an in vitro assay. As a control group, we have used patients with Alzheimer's disease, in which some patients may lose a considerable amount of weight, without an obvious cause. The serum lipolytic activity for the Alzheimer's group with weight loss (0.11 +/- 0.02 mumols glycerol released 10(5) adipocytes-1 ml-1 serum) was not significantly different from the group without weight loss (0.11 +/- 0.02 mumols glycerol released 10(5) adipocytes-1 ml-1) or from a healthy control group (0.07 +/- 0.02 mumols glycerol released 10(5) adipocytes-1 ml-1), but all three groups were significantly (P less than 0.005) lower than the cancer patient group (0.20 +/- 0.03 mumols glycerol 10(5) adipocytes-1 ml-1), irrespective of weight loss. A similar difference between the cancer and the control group was observed for the urinary lipolytic activity (0.67 +/- 0.03 versus 0.28 +/- 0.03 mumols glycerol released 10(5) adipocytes-1 mg creatinine-1 respectively, P less than 0.01). Weight loss in animals bearing the MAC16 adenocarcinoma was paralleled by a corresponding rise in serum lipolytic activity which peaked when the loss of carcass weight was 16%. A similar decrease in serum lipolytic activity was also observed in cancer patients at high percentages loss in body weight. However, a linear relationship was observed between both the serum and urinary lipolytic activity and weight loss in cancer patients (correlation coefficients 0.79 and 0.70 respectively) when the total body weight loss did not exceed 20%. This suggests that weight loss in cancer patients may be attributed, at least in part, to an, as yet, unidentified lipolytic factor.


Br. J. Cancer (1990), 62, 816-821 © Macmillan Press Ltd., 1990

### Alteration of serum and urinary lipolytic activity with weight loss in cachectic cancer patients

P. Groundwater[1], S.A. Beck[1], C. Barton[2], C. Adamson[1], I.N. Ferrier[3] & M.J. Tisdale[1]

[1]*Cancer Research Campaign Experimental Chemotherapy Group, Pharmaceutical Sciences Institute, Aston University, Birmingham B4 7ET;* [2]*CRC Clinical Trials Unit, Queen Elizabeth Hospital, Edgbaston, Birmingham B15 2TH; and* [3]*MRC Neurochemical Pathology Unit, Newcastle General Hospital, Newcastle upon Tyne NE4 6BE, UK.*

**Summary** The possibility that weight loss in cancer patients may be augmented by tumour produced catabolic factors, which stimulate lipid mobilisation, was investigated in a group of cancer patients with total body weight loss ranging from 0 to 50%. The serum and urine lipolytic activity has been determined using freshly isolated murine adipocytes in an *in vitro* assay. As a control group, we have used patients with Alzheimer's disease, in which some patients may lose a considerable amount of weight, without an obvious cause. The serum lipolytic activity for the Alzheimer's group with weight loss ($0.11 \pm 0.02\,\mu$mol glycerol released $10^5$ adipocytes$^{-1}$ ml$^{-1}$ serum) was not significantly different from the group without weight loss ($0.11 \pm 0.02\,\mu$mol glycerol released $10^5$ adipocytes$^{-1}$ ml$^{-1}$) or from a healthy control group ($0.07 \pm 0.02\,\mu$mol glycerol released $10^5$ adipocytes$^{-1}$ ml$^{-1}$), but all three groups were significantly ($P < 0.005$) lower than the cancer patient group ($0.20 \pm 0.03\,\mu$mol glycerol $10^5$ adipocytes$^{-1}$ ml$^{-1}$), irrespective of weight loss. A similar difference between the cancer and the control group was observed for the urinary lipolytic activity ($0.67 \pm 0.03$ versus $0.28 \pm 0.03\,\mu$mol glycerol released $10^5$ adipocytes$^{-1}$ mg creatinine$^{-1}$ respectively, $P < 0.01$). Weight loss in animals bearing the MAC16 adenocarcinoma was paralleled by a corresponding rise in serum lipolytic activity which peaked when the loss of carcass weight was 16%. A similar decrease in serum lipolytic activity was also observed in cancer patients at high percentages loss in body weight. However, a linear relationship was observed between both the serum and urinary lipolytic activity and weight loss in cancer patients (correlation coefficients 0.79 and 0.70 respectively) when the total body weight loss did not exceed 20%. This suggests that weight loss in cancer patients may be attributed, at least in part, to an, as yet, unidentified lipolytic factor.

Depletion of lipid stores is commonly found in cancer patients and may account for the largest part of the weight loss seen in cancer-bearing states (McAndrew, 1986). The effect appears unrelated to nutrient intake, since pair-fed animals do not lose as much fat as tumour-bearing animals (Lundholm *et al.*, 1981), and loss of body fat can occur in the absence of anorexia (Beck & Tisdale, 1987). An increased mobilisation of host adipose tissue may begin early in the development of the tumour (Beck & Tisdale, 1987; Kralovic *et al.*, 1977), and in tumours which produce cachexia is directly related to the tumour burden (Hollander *et al.*, 1986).

The mechanism for the increased mobilisation of host lipids in the tumour-bearing state is unknown, but may be related to the production of a lipolytic factor by the tumour cells. Loss of body fat can be produced by the injection of a non-viable preparation of Krebs-2-carcinoma, a tumour capable of causing extensive fat depletion in the host (Costa & Holland, 1962). Several lipolytic factors have been purified or partially purified from tumour cells. Thus a 75,000 Dalton protein, toxohormone L, has been isolated from tumour extracts and body fluids of patients and and animals (Masuno *et al.*, 1981), which, when injected into animals was capable of causing lipid mobilisation, immunosuppression and involution of the thymus gland (Masuno *et al.*, 1984). An enhanced lipid mobilisation was also produced by serum of mice bearing a thymic lymphoma (Kitada *et al.*, 1980). Serum from a patient with advanced cancer also produced a similar effect (Kitada *et al.*, 1981). Initial studies on this factor indicated a molecular weight of about 5,000 Daltons, while later studies showed that the low molecular weight form was inactive, but aggregated on standing in the cold to become active (Kitada *et al.*, 1982). Recently we have demonstrated a lipid mobilising factor in extracts of the MAC16 colon adenocarcinoma, a tumour capable of producing a 30% loss of host body weight with a tumour burden of only 3%, and without a drop in caloric intake (Beck & Tisdale, 1987). This material was also present in the serum suggesting a peripheral effect of the tumour. Lipid mobilising factors similar in charge and molecular weight to that found in the MAC16 tumour have recently also been identified in the serum of cachectic cancer patients.

These results suggest a generality of lipolytic factors in experimental cancer, but few studies have been carried out in patients. Production of lipolytic factors may be useful in the initial diagnosis of cancer patients and in determining the subsequent response to therapy. The level of such factors in body fluids of patients with various degrees of weight loss, with and without accompanying anorexia has been determined in the present study. As a control group we have used patients with senile dementia of the Alzheimer type in which patients may lose a considerable amount of weight with an apparently normal food intake (Sing *et al.*, 1988).

### Materials and methods

### *Subjects*

Twenty-four patients, 14 male and ten female with histologically proven malignancy and varying degrees of weight loss were entered into the study (Table I). None of the patients were receiving therapy at the time of serum and urine collection. Weight loss was calculated from the premorbid weight. Appetite and food intake scores were documented by the patients using a simple questionnaire and linear analogue scale. In addition, 19 patients with senile dementia of the Alzheimer type, ten with weight loss and nine without, were also entered into the study (Table I). The appetite and food intake of these patients was judged clinically. Seven normal laboratory workers served as disease-free controls. Blood samples were allowed to clot on ice for 60 min, centrifuged and the serum separated and divided into five aliquots. Samples were stored at − 70°C until assay. Urine was collected at the same time as blood was removed.

Correspondence: M.J. Tisdale.
Received 12 February 1990; and in revised form 21 May 1990.

**Table I** Characterics of patients used in the study[a]

| *Patient* | *Sex* | *Diagnosis* | *Weight loss (kg)* | *Duration of weight loss (weeks)* | *Total %[b] weight loss* | *Rate weight loss (kg/week)* | *State of weight loss[c]* | *Appetite score[d]* | *Intake score[e]* |
|---|---|---|---|---|---|---|---|---|---|
| 1 | M | Lung cancer (squamous) | 12 | 11 | 17 | 1.06 | Progressive | 2 | 1 |
| 2 | M | Oesophageal cancer | 57–64 | 24 | 50 | 2.67 | Static | 2 | 2 |
| 3 | F | Ovarian cancer | 17 | 21 | 15 | 0.82 | Progressive | 3 | 3 |
| 4 | F | Lung cancer (oat) | 24 | 52 | 36 | 0.46 | Progressive | 1 | 1 |
| 5 | F | Breast cancer | 0 | 0 | 0 | 0 | – | 3 | 3 |
| 6 | F | Colonic cancer | 24 | 48 | 36 | 0.5 | Progressive | 2 | 2 |
| 7 | F | Non Hodgkins lymphoma | 2 | 7 | 4 | 0.29 | Progressive | 2 | 2 |
| 8 | M | Hodgkins disease | 0 | 0 | 0 | 0 | – | 3 | 3 |
| 9 | M | Myeloma | 0 | 0 | 0 | 0 | – | 3 | 3 |
| 10 | M | Lung cancer (squamous) | 9 | 52 | 18 | 0.17 | Progressive | 1 | 2 |
| 11 | M | Renal cancer | 8[f] | 13 | 11 | 0.60 | Progressive | 2 | 3 |
| 12 | F | Liposarcoma | 27 | 52 | 28 | 0.51 | Progressive | 3 | 2 |
| 13 | M | Renal cell carcinoma | 2 | 5 | 9 | 0.46 | Progressive | 1 | 2 |
| 14 | F | Cervical cancer | 3 | 10 | 25 | 0.34 | Progressive | 2 | 2 |
| 15 | M | Non Hodgkins lymphoma | 5 | 12 | 6 | 0.38 | Progressive | 2 | 4 |
| 16 | M | Malignant teratoma | 5 | 6 | 6 | 0.83 | Progressive | 2 | 3 |
| 17 | F | Lung cancer (squamous) | 5 | 5 | 6 | 0.9 | Static | 3 | 3 |
| 18 | M | Lung cancer (squamous) | 4 | 8 | 8 | 0.5 | Progressive | 2 | 1 |
| 19 | F | Breast cancer | 7 | 81 | 14 | 0.08 | Progressive | 3 | 3 |
| 20 | M | Non Hodgkins lymphoma | 7 | 20 | 10 | 0.35 | Progressive | 2 | 2 |
| 21 | M | Non Hodgkins lymphoma | 18[f] | 8 | 20 | 2.09 | Progressive | 2 | 1 |
| 22 | F | Breast cancer | 14 | 22 | 18 | 0.64 | Progressive | 2 | 2 |
| 23 | M | Malignant teratoma | 3 | 3 | 3 | 0.87 | Progressive | 2 | 2 |
| 24 | M | Gastric carcinoma | 32 | 26 | 43 | 1.2 | Progressive | 1 | 1 |
| 25 | F | Alzheimer's disease | 10 | 38 | 17 | 0.26 | Progressive | 3 | 3 |
| 26 | F | Alzheimer's disease | 22 | – | 39 | – | Progressive | 3 | 3 |
| 27 | F | Alzheimer's disease | 17 | 70 | 26 | 0.24 | Progressive | 3 | 3 |
| 28 | F | Alzheimer's disease | 8 | – | 17 | – | Progressive | 3 | 3 |
| 29 | F | Alzheimer's disease | 15 | – | 27 | – | Progressive | 3 | 3 |
| 30 | F | Alzheimer's disease | 8 | 44 | 18 | 0.18 | Progressive | 3 | 3 |
| 31 | F | Alzheimer's disease | 6.5 | 72 | 12 | 0.09 | Progressive | 3 | 3 |
| 32 | F | Alzheimer's disease | 37 | 8 | 45 | 4.63 | Progressive | 3 | 3 |
| 33 | F | Alzheimer's disease | 11 | 40 | 20 | 0.28 | Progressive | 3 | 3 |
| 34 | F | Alzheimer's disease | 6.5 | 72 | 14 | 0.08 | Progressive | 3 | 3 |
| 35 | F | Alzheimer's disease | – | – | – | – | – | – | – |
| 36 | F | Alzheimer's disease | – | – | – | – | – | – | – |
| 37 | F | Alzheimer's disease | – | – | – | – | – | – | – |
| 38 | F | Alzheimer's disease | – | – | – | – | – | – | – |
| 39 | F | Alzheimer's disease | – | – | – | – | – | – | – |
| 40 | F | Alzheimer's disease | – | – | – | – | – | – | – |
| 41 | F | Alzheimer's disease | – | – | – | – | – | – | – |
| 42 | F | Alzheimer's disease | – | – | – | – | – | – | – |
| 43 | F | Alzheimer's disease | – | – | – | – | – | – | – |
| 44 | M | Normal | – | – | – | – | – | – | – |
| 45 | F | Normal | – | – | – | – | – | – | – |
| 46 | F | Normal | – | – | – | – | – | – | – |
| 47 | F | Normal | – | – | – | – | – | – | – |
| 48 | F | Normal | – | – | – | – | – | – | – |
| 49 | M | Normal | – | – | – | – | – | – | – |
| 50 | F | Normal | – | – | – | – | – | – | – |

[a]None of the patients was receiving therapy at the time of the study. [b]Weight loss as percentage of weight from which the weight loss is calculated (usually, but not always, the pre-morbid weight). [c]Some patients may have lost significant weight, but not over the last few weeks. [d]1 = no appetite, 2 = less than usual, 3 = usual, 4 = more than usual, 5 = much more than usual, as determined by patient. [e]1 = much less than usual food intake, 2 = less than usual, 3 = usual, 4 = more than usual, 5 = much more than usual, as determined by patient. [f]Ascites present: weight loss probably an underestimate.

### *Animal studies*

Female NMRI mice (obtained from our own colony) average weight 20 g were transplanted with fragments of the MAC16 tumour into the flank as previously described (Beck & Tisdale, 1987). At 14 days after transplantation, when weight loss was expected, blood was removed from the tail vein at daily intervals. Body weight was also measured daily. Under Home Office regulations, the experiment was terminated when weight loss reached 30% of the initial body weight. Plasma was prepared by centrifuging whole blood in a Beckman microfuge for 30s.

### *Determination of lipolytic activity*

Mice (strain BKW) were killed by cervical dislocation and their epididymal adipose tissue was removed and placed in isotonic saline, minced and incubated at 37°C for 2 h in Krebs Ringer bicarbonate buffer, pH 7.2, containing 2 mg $ml^{-1}$ of collagenase (Sigma Chemical Co., Dorset, UK) with prior gassing with 95%$O_2$:5%$CO_2$. Digestion of the tissue was detected by the disappearance of intact pieces and an increased turbidity of the medium. Undigested material and non-adipose matter was removed by allowing the fat cells to float to the surface of the buffer and the supernatant was aspirated and replaced with fresh buffer. The washing procedure was repeated three times to remove all collagenase, non-adipose cells and any endogenous hormones. After the final wash the cells were suspended in an appropriate amount of Krebs Ringer solution to give a density of $1.5 \times 10^5$ adipocytes $ml^{-1}$, the cell number being enumerated with a Neubauer haemocytometer.

Cell samples (1 ml) were removed, with continuous mixing to maintain a homogeneous cell suspension, added to the appropriate test substance, gassed with 95%$O_2$:5%$CO_2$ and incubated for 2 h at 37°C in a shaking water bath. Control samples containing adipocytes alone were also analysed to measure any spontaneous glycerol release. When assaying serum samples, a control (no adipocytes) was also included to measure the initial amount of glycerol present in the serum. Routinely samples of serum and urine (100 µl) were assayed in duplicate and the assay was repeated four to five times on each sample at different times using the separately stored aliquots. At the end of the incubation period, 0.5 ml of the incubation buffer was added to 0.5 ml of 10% (w/v) perchloric acid and the mixture was shaken to ensure deproteinisation. The precipitated protein was sedimented by centrifugation at 2,000 r.p.m. for 10 min, the supernatant removed and neutralised with 20% (w/v) KOH, after which the potassium perchlorate was sedimented by centrifugation (2,000 r.p.m., 10 min) and the volume of the supernatant was recorded and used to calculate the dilution factor. Assays on the supernatant were performed either immediately, or after storage at − 20°C for between 18 and 72 h. The concentration of glycerol was determined enzymatically on 200 µl aliquots of the supernatant by the method of Wieland (1974). The results are expressed as µmol glycerol released per ml of serum or per mg creatinine in urine per $10^5$ adipocytes minus both the fat cell control value and the serum or urine control value. Urinary creatinine concentration was determined colorimetrically at 500 nm using a Sigma diagnostic kit (Sigma Diagnostic, Poole, Dorset, UK).

### *Statistical analysis*

Results are expressed as mean ± s.e.m. for at least five separate determinations on a single patient when samples were available. Differences were determined statistically using Student's *t* test.

### Results

The characteristics of the patients in this study are shown in Table I. Total percentage body weight loss varied between 0 and 50% and the rate of weight loss varied between 0 and 4.6 kg per week. The average weight loss in the cancer patient group (13.5 ± 2.9 kg) was not significantly different from that in the Alzheimer's group (14.0 ± 3.0 kg). Of the 21 evaluable cancer patients with weight loss, 16 (76%) reported a decrease in appetite and 15 (71%) reported a decreased food intake. Of these 15 only five (36%) reported a much greater decreased food intake than normal. All of the patients with cancer, but with no weight loss, had normal appetite and food intake scores. In general cancer patients who reported a normal food intake had a lower rate of weight loss (range 0–0.9 kg per week) compared with patients with a reduced intake score (range 0.17–2.7 kg per week). Both the appetite and intake scores were irrespective of tumour type.

**Figure 1** Dose–response relationship for the ability of isoprenaline to stimulate lipolysis, as measured by release of FFA, from murine epididymal adipocytes. The concentration of FFA in cell-free supernatants was determined immediately after incubation using a Wako NEFA C kit (Alpha Laboraties Ltd., Hampshire, UK) as previously described (Beck & Tisdale, 1987).

### *Serum lipolytic activity*

The rate of glycerol release from murine adipocytes in response to both serum and urine samples increased linearly over a 3 h period, but tended to decrease with increasing time of incubation, and for this reason 2 h was chosen as a convenient incubation perioid for all samples. At the 2 h time point there was a doubling in the amount of glycerol released when the sample volume was increased from 50 to 100 µl, but the linearity did not extend above this range. For this reason, and to obtain a larger change in absorbence value, the sample volume was restricted to 100 µl for both urine and serum. The serum and urine glycerol content was subtracted from the final figures. In the case of urine samples this value

**Table II** Serum and urine lipolytic activity in cancer patients[a]

| Patient | *Serum lipolytic activity µmol glycerol released $10^5$ adipocytes$^{-1}$ ml$^{-1}$* | *Urine lipolytic activity µmol glycerol released $10^5$ adipocytes$^{-1}$ mg creatinine$^{-1}$* |
|---|---|---|
| 1 | 0.36 ± 0.03 | 1.02 ± 0.04 |
| 2 | 0.09 ± 0.01 | 0.50 ± 0.05 |
| 3 | 0.14 ± 0.03 | 0.94 ± 0.03 |
| 4 | 0.18 ± 0.03 | 0.23 ± 0.03 |
| 5 | 0.22 ± 0.02 | 0.56 ± 0.06 |
| 6 | 0.12 ± 0.01 | 0.83 ± 0.07 |
| 7 | 0.20 ± 0.04 | 0.57 ± 0.02 |
| 8 | 0.16 ± 0.01 | 0.33 ± 0.03 |
| 9 | 0.12 ± 0.01 | 0.31 ± 0.02 |
| 10 | 0.22 ± 0.04 | 0.60 ± 0.12 |
| 11 | 0.31 ± 0.03 | 0.28 ± 0.02 |
| 12 | 0.12 ± 0.01 | 0.45 ± 0.06 |
| 13 | 0.12 ± 0.02 | 0.27 ± 0.01 |
| 14 | 0.11 ± 0.06 | 0.40 ± 0.10 |
| 15 | 0.23 ± 0.04 | 1.10 ± 0.08 |
| 16 | 0.22 ± 0.04 | 0.46 ± 0.02 |
| 17 | 0.11 ± 0.03 | 0.38 ± 0.06 |
| 18 | 0.09 ± 0.02 | 0.18 ± 0.03 |
| 19 | 0.10 ± 0.02 | 1.11 ± 0.04 |
| 20 | 0.33 ± 0.02 | 0.63 ± 0.02 |
| 21 | 0.20 ± 0.02 | 0.48 ± 0.02 |
| 22 | 0.63 ± 0.00 | 1.22 ± 0.04 |
| 23 | 0.12 ± 0.006 | 0.25 ± 0.06 |
| 24 | 0.26 ± 0.01 | 0.81 ± 0.05 |
| 25 | 0.10 ± 0.02 | – |
| 26 | 0.11 ± 0.01 | – |
| 27 | 0.10 ± 0.05 | – |
| 28 | 0.17 ± 0.07 | – |
| 29 | 0.00 ± 0.00 | – |
| 30 | 0.16 ± 0.04 | – |
| 31 | 0.07 ± 0.01 | – |
| 32 | 0.06 ± 0.01 | – |
| 33 | 0.12 ± 0.05 | – |
| 34 | 0.16 ± 0.00 | – |
| 35 | 0.16 ± 0.08 | – |
| 36 | 0.13 ± 0.01 | – |
| 37 | 0.13 ± 0.07 | – |
| 38 | 0.06 ± 0.00 | – |
| 39 | 0.01 ± 0.00 | – |
| 40 | 0.18 ± 0.01 | – |
| 41 | 0.14 ± 0.03 | – |
| 42 | 0.07 ± 0.02 | – |
| 43 | 0.14 ± 0.07 | – |
| 44 | 0.09 ± 0.002 | 0.09 ± 0.02 |
| 45 | 0.00 ± 0.00 | 0.50 ± 0.09 |
| 46 | 0.13 ± 0.01 | 0.59 ± 0.08 |
| 47 | 0.02 ± 0.001 | 0.09 ± 0.01 |
| 48 | 0.01 ± 0.001 | 0.09 ± 0.01 |
| 49 | 0.10 ± 0.03 | 0.09 ± 0.02 |
| 50 | 0.12 ± 0.03 | 0.50 ± 0.09 |

[a]Results are given as mean ± s.e.m. for at least five separate determinations from individual patients, each determination being performed in triplicate. Where single values are given insufficient sample was available in separate aliquots for repeat experiments.

tended to be zero. The validity of the assay was established using isoprenaline as a positive control (Figure 1).

The serum lipid mobilising activity of patients with cancer, Alzheimer's disease and normal healthy controls is given in Table II. Preliminary experiments revealed that repeated freezing and thawing of serum samples resulted in a progressive loss of lipolytic activity. For this reason fresh serum samples were divided into aliquots before freezing and only the values obtained with freshly thawed samples are reported. The average serum lipolytic activity for the Alzheimer's group with weight loss ($0.11 \pm 0.02\,\mu$mol glycerol $10^5$ adipocytes$^{-1}$ ml$^{-1}$ serum) was not significantly different from the group without weight loss ($0.11 \pm 0.02\,\mu$mol glycerol $10^5$ adipocytes$^{-1}$ ml serum) or from the healthy control group ($0.07 \pm 0.02\,\mu$mol glycerol $10^5$ adipocytes$^{-1}$ ml$^{-1}$ serum), but all three groups were significantly lower ($P < 0.005$) than the cancer patient group ($0.20 \pm 0.03\,\mu$mol glycerol $10^5$ adipocytes$^{-1}$ ml$^{-1}$ serum) irrespective of whether weight loss was apparent. Patients with cancer, but with no weight loss also had a significantly higher serum lipolytic activity ($0.17 \pm 0.03\,\mu$mol glycerol $10^5$ adipocytes$^{-1}$ ml$^{-1}$ serum) than the Alzheimer's group or healthy controls ($P < 0.001$).

To try to understand variations in serum lipolytic activity with weight loss, measurements have been made in animals bearing the MAC16 adenocarcinoma, an experimental model of cachexia. Animals transplanted with this tumour are a heterogeneous group with weight loss appearing at various times after tumour transplantation. Occasionally animals bearing this tumour do not develop weight loss, although the growth of the tumour is similar to those in which weight loss is apparent. The results in Figure 2a show that for animals in which weight loss occurs there is a rise in the plasma level of lipolytic activity, which reaches a maximum when the animal has lost 16% of the body weight (Figure 2b) and thereafter decreases. Values of plasma lipolytic activity at all time points from day 4 to day 7 are significantly ($P < 0.01$) higher than non-tumour-bearing controls. For animals without weight loss there is no significant elevation in plasma lipolytic activity above the value found in non tumour-bearing controls (Figure 2c). This suggests a direct correlation between the rise in plasma lipolytic activity and the weight loss, although the relationship is only linear for the initial weight loss.

A similar relationship between serum lipolytic activity and weight loss was observed with the cancer patient group. Here there was a linear relationship between serum lipolytic activity and weight loss (correlation coefficient 0.79, $n = 18$) only when the total loss of body weight did not exceed 20% and weight loss was progressive (Figure 3). Patients with higher percentage loss of body weight tended to have low levels of serum lipolytic activity, and these patients all reported a decrease in food intake, while a number of patients with lower weight loss reported a normal food intake. When only these subjects were included, the correlation between serum lipolytic activity and weight loss improved slightly ($r = 0.84$, $n = 12$) (Figure 4).

Patients with previous weight loss who were stable during the study period had low levels of serum lipolytic activity (patients two and 19 with values of $0.09 \pm 0.01$ and $0.10 \pm 0.003\,\mu$mol glycerol released $10^5$ adipocytes$^{-1}$ ml$^{-1}$ serum respectively) that was not elevated significantly above the controls.

**Figure 2** Changes in serum lipolytic activity (□) with weight loss (●) in **a**, animals bearing the MAC16 tumour which lost weight, **b**, variation in serum lipolytic activity of animals in **a** with change in carcass weight, **c**, animals bearing the MAC16 tumour, but without weight loss. Results are expressed as mean ± s.e.m. for four animals per group. Lipolytic activity is expressed as $\mu$mol glycerol released per $10^5$ adipocytes per ml plasma in a 2 h incubation. The average value for non-tumour-bearing controls was $0.5 \pm 0.2\,\mu$mol glycerol $10^5$ adipocytes$^{-1}$ ml$^{-1}$.

*Urine lipolytic activity*

In general the values for the urine lipolytic activity were qualitatively in line with the serum values (Table II). However, the accuracy of detection was determined by the total volume of urine excreted since too dilute samples were below the limits of glycerol measurement. To counteract differences in urine volumes the urinary lipolytic activity has been expressed relative to the creatinine concentration of urine. The urine lipolytic activity for all cancer patients ($0.67 \pm 0.03\,\mu$mol glycerol released $10^5$ adipocytes$^{-1}$ mg creatinine$^{-1}$ urine) was significantly elevated above normal controls ($0.28 \pm 0.03\,\mu$mol glycerol released $10^5$ adipocytes$^{-1}$ mg creatinine$^{-1}$; $P < 0.01$). However, there was no significant difference in the non-weight losing cancer patients and controls unlike the serum value. A similar biphasic rise and fall of lipolytic activity with weight loss was observed. However, when only patients with weight loss less than 20% body weight were considered there was a linear relationship ($r = 0.70$, $n = 17$) between the urine lipolytic activity and weight loss for all patients (Figure 5). There was no correlation between the urinary lipolytic activity and the rate of weight loss.

**Figure 3** Relationship between serum lipolytic activity and weight loss in a group of cancer patients. The lipolytic activity is expressed as μmol glycerol released per $10^5$ adipocytes in a 2 h incubation per ml serum.

**Figure 4** Relationship between serum lipolytic activity and weight loss in a group of cancer patients reporting a normal food intake.

**Figure 5** Relationship between urinary lipolytic activity and weight loss in a group of cancer patients. The lipolytic activity is expressed as μmol glycerol released per $10^5$ adipocytes in a 2 h incubation per mg urinary creatinine.

### Discussion

A rapid reduction in the total quantity of body fat has been demonstrated in patients with cancer (Watkin, 1959), accompanied by hypermetabolism and an increased utilisation of fat. Diminished insulin secretion and resistance has been considered to enhance lipid mobilisation in cancer cachexia (Argiles & Azcon-Bieto, 1988), but the present study confirms the existence of an as yet unidentified lipolytic factor in the serum and urine of cancer patients with or without weight loss. This factor lacks species specificity in that the human material is able to stimulate lipolysis in murine adipocytes.

In animals bearing the MAC16 adenocarcinoma, an experimental tumour inducing weight loss in some, but not all recipient animals, plasma levels of lipolytic activity were only elevated in animals in which weight loss occurred. This suggests a direct correlation between this lipid mobilising factor and the induction of weight loss, and this correlation has been strengthened by the observation that the polyunsaturated fatty acid, eicosapentaenoic acid, is an inhibitor of both the tumour lipid mobilising factor and the weight loss in animals bearing the MAC16 tumour (Tisdale & Beck, unpublished). In animals bearing the MAC16 tumour there is only a linear correlation between the plasma level of the lipolytic factor and weight loss, when the total loss of body weight did not exceed 16%. Above this level plasma levels of lipolytic activity decreased with increasing weight loss. The mechanism of regulation of serum lipid mobilising activity is not known.

We have utilised freshly prepared murine adipocytes to investigate the lipolytic activity of patient samples in preference to slices of adipose tissue or cultured adipocytes. Free fat cells generally respond to all hormones which affect intact tissues and have a greater lipolytic response than pieces of adipose tissue incubated *in vitro* (Fair, 1973). The response of different preparations of adipocytes is somewhat variable and therefore we have carried out the experiments on at least five different occasions where sample volume permitted. The response of the adipocyte preparations has been standardised using isoprenaline as a positive control. All fat cell preparations release glycerol in the absence of a lipolytic agent. This blank figure is somewhat variable due to differences in the adipocyte preparation, but is subtracted from the final figures. The values for the lipolytic activity of serum samples decreased on repeated freezing and thawing and therefore only values are included where the sample was not re-frozen. In some cases this did not give sufficient results for statistical analysis.

While the serum lipolytic activity of the cancer patient group was significantly elevated over that of healthy controls, patients with Alzheimer's disease and weight loss comparable to that found in the cancer patients did not differ either from Alzheimer's patients without weight loss or from healthy controls. This suggests that elevation of serum lipolytic activity is not common in all weight losing situations and may be specific for the neoplastic state.

When only patients with weight loss less than 20% of body weight are considered there is a good correlation between the level of the serum and urinary lipolytic activity and the extent of weight loss, as for the MAC16 tumour. Patients with weight loss greater than 20% of body weight have low serum levels of lipolytic activity and in these patients anorexia was invariably present. This suggests that a tumour-produced lipolytic factor may be important in weight loss, particularly when anorexia is absent.

Anorexia is commonly reported in cancer patients (Bernstein, 1986) and in our study a high percentage (70%) of the weight losing cancer patients, reported a decrease in appetite and food intake. This was, however, only a subjective assessment by the patients and anorexia is sometimes denied by the patient even though it may be present (Wesdorp *et al.*, 1986). However, detailed studies have shown that the energy intake in ten cancer patients, who were below normal body weight and body cell mass, did not differ significantly from that of nine non-neoplastic control subjects, with diseases affecting physical activity to about the same extent (Warnold, *et al.*, 1978). Both the daily energy expenditure and the resting metabolic rate were significantly greater in the cancer patients than in the controls. Since lipids have a high calorific value, they are probably important in maintaining the high metabolic rate in cancer patients, and an increased lipid requirement in cancer patients is suggested, since the rate of removal of infused lipids from the blood appears to be increased (Waterhouse & Nye, 1961). Thus a lipolytic factor may be important in cancer patients for providing increased lipid mobilisation under conditions where it might not be expected to occur, e.g. when the energy intake is normal.

It is also known that a number of tumours have a limited ability to synthesise fatty acids and obtain a substantial amount preformed from the host (Spector & Burns, 1987). Such fatty acids after only minor structural modification are incorporated into all of the complex lipids formed by the tumour cells, including the phospholipids needed for membrane synthesis and the formation of important regulatory metabolites such as eicosanoids and diacylglycerol. Thus the elaboration of a lipolytic factor may be essential for the growth and reproduction of neoplastic cells. However, measurement of the rate of whole body lipolysis in man using the glycerol turnover rate has shown no difference between cancer patients and controls (Jeevanandam *et al.*, 1986), although another study using the same technique reported an elevated glycerol turnover in progressive cancer (Eden *et al.*, 1985). Thus, whether the loss of body fat in patients with cancer cachexia is due to a reduced rate of lipogenesis or an augmented lipolysis still remains controversial, although our results would support the latter hypothesis.

The only urinary lipolytic factor so far described in humans was obtained from fasting subjects with intact pituitaries (Kekwick & Pawan, 1967) and has many of the characteristics of a cachectic factor. Thus when injected into mice this material mobilises body fat, increases the total metabolic turnover and causes weight loss without depressing appetite (Chalmers *et al.*, 1958). Another postulated cachectic factor, tumour necrosis factor/chachectin (TNF) produced by activated macrophages, may be correlated with appetite suppression and anorexia, but has no direct lipolytic activity in an *in vitro* assay (Mahony *et al.*, 1988). Furthermore, using a sensitive radioimmunoassay TNF was not detectable in the serum of patients with clinical cancer cachexia (Socher *et al.*, 1988). For this reason no estimation of the levels of TNF has been made in the present study.

The nature of the serum and urinary lipolytic factor found in the present study awaits its purification although preliminary results suggest that the material differs from the lipolytic hormones found in normal serum and is similar to the lipid mobilising factor elaborated by the cachexia-inducing MAC16 adenocarcinoma in both charge and molecular weight. However, the elevated levels found in cancer patients may be a useful aid in the initial diagnosis of the disease and further studies will determine the response to therapy.

This work has been supported by a grant from the Cancer Research Campaign. S.A.B. gratefully acknowledges the receipt of a research studentship from the Cancer Research Campaign. We would like to thank Sister D. Lett and the staff of St Nicholas Hospital, Newcastle upon Tyne.

### References

ARGILES, J.M. & AZCON-BIETO, J. (1988). The metabolic environment of cancer. *Mol. Cell Biochem.*, **81**, 3.

BECK, S.A. & TISDALE, M.J. (1987). Production of lipolytic and proteolytic factors by a murine tumor-producing cachexia in the host. *Cancer Res.*, **47**, 5919.

BERNSTEIN, I.L. (1986). Etiology of anorexia in cancer. *Cancer*, **58**, 1881.

CHALMERS, T.M., KEKWICK, A. & PAWAN, G.L.S. (1958). On the fat-mobilising activity of human urine. *Lancet*, **i**, 866.

COSTA, G. & HOLLAND, J.F. (1962). Effects of Krebs-2 carcinoma on the lipid metabolism of male Swiss mice. *Cancer Res.*, **22**, 1081.

EDEN, E., EDSTRÖM, S. & BENNEGARD, K. (1985). Glycerol dynamics in weight-losing cancer patients. *Surgery*, **97**, 176.

FAIR, J.N. (1973). Biochemical aspects of drug and hormone action on adipose tissue. *Pharmacol Rev.*, **25**, 67.

HOLLANDER, D.M., EBERT, E.C., ROBERTS, A.I. & DEVEREUX, D.F. (1986). Effects of tumor type and burden on carcass lipid depletion in mice. *Surgery*, **100**, 292.

JEEVANANDAM, M., HOROWITZ, G.D., LOWRY, S.F. & BRENNAN, M.F. (1986). Cancer cachexia and the rate of whole body lipolysis in man. *Metabolism*, **35**, 304.

KEKWICK, A. & PAWAN, G.L.S. (1967). Fat mobilizing substance. *Metabolism*, **16**, 787.

KITADA, S., HAYS, E.F. & MEAD, J.F. (1980). A lipid mobilizing factor in serum of tumor-bearing mice. *Lipids*, **15**, 168.

KITADA, S., HAYS, E.F. & MEAD, J.F. (1981). Characterization of lipid mobilizing factor from tumors. *Prog. Lipid Res.*, **28**, 823.

KITADA, S., HAYS, E.F., MEAD, J.F. & ZABIN, I. (1982). Lipolysis induction in adipocytes by a protein from tumor cells. *J. Cell Biochem.*, **20**, 409.

KRALOVIC, R.C., ZEPP, E.A. & CENEDELLA, R.J. (1977). Studies on the mechanism of carcass fat depletion in experimental cancer. *Eur. J. Cancer*, **13**, 1071.

LUNDHOLM, K., EDSTRÖM, S., EKMAN, L., KARLBERG, I. & SCHERSTEN, T. (1981). Metabolism in peripheral tissues in cancer patients. *Cancer Treat. Rep.*, **65** (Suppl. 5), 79.

MAHONY, S.M., BECK, S.A. & TISDALE, M.J. (1988). Comparison of weight loss induced by recombinant tumour necrosis factor with that produced by a cachexia-inducing tumour. *Br. J. Cancer*, **57**, 385.

MASUNO, H., YAMASAKI, N. & OKUDA, H. (1981). Purification and characterization of lipolytic factor (toxohormone-L) from cell-free fluid of ascites sarcoma 180. *Cancer Res.*, **41**, 284.

MASUNO, H., YOSHIMURA, H., OGAWA, N. & OKUDA, H. (1984). Isolation of lipolytic factor (Toxohormone-L) from ascites fluid of patients with hepatoma and its effect on feeding behaviour. *Eur. J. Cancer Clin. Oncol.*, **20**, 1177.

MCANDREW, P.F. (1986). Fat metabolism and cancer. *Surg. Clin. North Am.*, **66**, 1003.

SINGH, S., MULLEY, G.P. & LOSOWSKY, M.S. (1988). Why are Alzheimer patients thin? *Age Ageing*, **17**, 21.

SOCHER, S.H., MARTINEZ, D., CRAIG, J.B., KUHN, J.G. & OLIFF, A. (1988). Tumor necrosis factor not detectable in patients with clinical cancer cachexia. *J. Natl Cancer Inst.*, **80**, 595.

SPECTOR, A.A. & BURNS, C.P. (1987). Biological and therapeutic potential of membrane lipid modification in tumors. *Cancer Res.*, **47**, 4529.

WARNOLD, I., LUNDHOLM, K. & SCHJERSTEN, T. (1978). Energy balance and body composition in cancer patients. *Cancer Res.*, **38**, 1801.

WATERHOUSE, C. & NYE, W.H.R. (1961). Metabolic effects of infused triglyceride. *Metabolism*, **10**, 403.

WATKIN, D.M. (1959). Increased fat utilization in the hypermetabolism of active neoplastic disease. *Acta Un. Int. Cancer*, **15**, 907.

WESDORP, R.I.C., KRAUSE, R. & VON MYENFELDT, M.F. (1986). Cancer cachexia and its nutritional implications. *Br. J. Surg.*, **70**, 352.

WIELAND, O. (1974). Glycerol UV method. In *Methods of Enzymatic Analysis, 3*, Bergemeyer, H.U. (ed.) p. 1404. Academic Press: London.